# Evaluating the Effects of the Supportive Parenting App on Infant Developmental Outcomes: Longitudinal Study

**DOI:** 10.2196/43885

**Published:** 2023-02-22

**Authors:** Shefaly Shorey, Yap Seng Chong, Luming Shi, Jing Shi Chua, Thilagamangai, Jancy Mathews, Siew Hoon Lim, Ruochen Du, Yiong Huak Chan, Thiam Chye Tan, Cornelia Chee, Evelyn Law

**Affiliations:** 1 Alice Lee Centre for Nursing Studies National University of Singapore Singapore Singapore; 2 Yong Loo Lin School of Medicine National University of Singapore Singapore Singapore; 3 Singapore Clinical Research Institute Singapore Singapore; 4 Division of Nursing KK Women’s and Children’s Hospital Singapore Singapore; 5 National University Polyclinics Singapore Singapore; 6 Singapore General Hospital Singapore Singapore; 7 Mount Elizabeth Novena Specialist Centre Singapore Singapore; 8 National University Hospital Singapore Singapore

**Keywords:** infant development, parenting, mobile health technology, social support, psychoeducation, peer support, mobile phone

## Abstract

**Background:**

Previous studies have investigated the various effects of parenting on infant developmental outcomes. In particular, parental stress and social support have been found to significantly affect the growth of the newborn. Although many parents today use mobile apps to obtain more support in parenting and perinatal care, few studies have examined how these apps could affect infant development.

**Objective:**

This study aimed to examine the effectiveness of the Supportive Parenting App (SPA) in improving infant developmental outcomes during the perinatal period.

**Methods:**

This study adopted a 2-group parallel prospective longitudinal design and recruited 200 infants and their parents (N=400 mothers and fathers). The parents were recruited at 24 weeks of gestation for a randomized controlled trial conducted from February 2020 to July 2022. They were randomly allocated to either the intervention or control group. The infant outcome measures included cognition, language, motor skills, and social-emotional development. Data were collected from the infants when they were aged 2, 4, 6, 9, and 12 months. Linear and modified Poisson regressions were used to analyze the data to examine between- and within-group changes.

**Results:**

At 9 and 12 months post partum, the infants in the intervention group were found to have better communication and language skills than those in the control group. An analysis of motor development revealed that a larger proportion of the infants in the control group fell under the at-risk category, where they scored approximately 2 SDs below the normative scores. The control group infants scored higher on the problem solving domain at 6 months post partum. However, at 12 months postpartum, the infants in the intervention group performed better on cognitive tasks than those in the control group. Despite not being statistically significant, the intervention group infants were found to have consistently scored better on the social components of the questionnaires than the control group infants.

**Conclusions:**

Overall, the infants whose parents had received the SPA intervention tended to fare better in most developmental outcome measures than those whose parents had received standard care only. The findings of this study suggest that the SPA intervention exerted positive effects on the communication, cognition, motor, and socioemotional development of the infants. Further research is needed to improve the content and support provided by the intervention to maximize the benefits gained by infants and their parents.

**Trial Registration:**

ClinicalTrials.gov NCT04706442; https://clinicaltrials.gov/ct2/show/NCT04706442

## Introduction

### Background

The effects of parenting on infant development are a widely investigated topic. Studies have found that parenting knowledge, parental stress, and parental perceived support have significant impacts on the growth of an infant [1-3]. For example, parents’ knowledge of and participation in their child’s play contribute significantly to the development of the child, as it helps boost executive functioning, encourage prosocial behavior, and enhance creativity [4]. Greater parenting stress and low levels of perceived social support have also been found to be associated with depression among mothers, which is correlated with developmental delays in infants [1].

The lack of social support has been linked to various parental outcomes, such as postnatal depression [5], parental stress [6], and anxiety [7]. Social support is often regarded as a protective factor for parents, especially during the perinatal period. Receiving support from others often helps parents feel less overwhelmed, aiding them with the transition to parenthood or helping them cope with having to care for multiple children. Some studies [8-10] conducted in Singapore found that parents desire more informational and familial support. Specifically, some parents perceived having a lack of knowledge of infant care and wanted to have access to reliable information sources [8]. With the rapid advancements in technology over the last 2 decades, parents today tend to look for information from web-based sources or seek support from web-based parenting communities, as these sources are extremely convenient and accessible [10-12]. They are mostly aware that information found on the web can be fabricated or exaggerated [12]; therefore, they tend to prefer gathering information directly from health care professionals such as obstetricians and neonatologists [8]. This is not always possible, especially during the postnatal period, as parents no longer have regular appointments with their obstetricians or gynecologists. As a result, parents try to obtain accurate information on the web by visiting only reputable sites that disseminate information provided by health care professionals or going to less commercially based websites [13]. Even then, these sites might not be consistent in the information they provide, especially when the information is not contextualized and does not incorporate cultural norms, and this may cause confusion among parents regarding what the right childcare practices are [14]. This justifies the need to create evidence-based programs tailored to the present generation of tech-savvy parents to improve their well-being and aid them in developing competent childcare skills [15].

According to Milgrom et al [5], it is recommended to implement programs to improve parental well-being during the perinatal period, as social support plays a large role in mediating the relationship between postnatal depression and child development during this time. During the perinatal period, low levels of social support such as insufficient partner support [5], lack of reliable information sources [16], and caring for a newborn without aid from others [17] often induce much stress and negative moods in parents. To fulfill the support needs of parents during the perinatal period, a mobile health (mHealth) app known as the Supportive Parenting App (SPA) was developed. The mHealth SPA was developed as a one-stop resource center because past studies have found technology-based interventions to be effective in offering parenting education and support [18,19]. Such remote interventions were found to be helpful specifically for parents who were facing childcare issues but were not always able to seek immediate advice from health care professionals [18,19].

The SPA is a theory- and evidence-based psychoeducational app developed using different theoretical frameworks, such as Singh et al’s [20] mHealth user engagement pyramid, Bandura’s [21] social cognitive theory, and Bowlby’s [22] attachment theory. Through SPA, parents were able to obtain information on childcare and parenting-related topics to aid them in their parenting journey. In addition, unique to SPA, a peer support feature was included to provide parents with emotional support from trained peer volunteers. Although various parenting interventions have been developed and evaluated to improve parental outcomes, a recent review by Adina et al [23] found that few studies have explored how these interventions can indirectly affect infant or child developmental outcomes. This is unexpected, as the improvement of child developmental outcomes is often cited as a reason for developing these parenting programs [1,15]. Therefore, although these interventions are often directed at parents, it is important to examine how the development of infants may be affected as a consequence.

### Aims and Hypotheses

This study aimed to examine whether the SPA intervention had any indirect effects on the developmental outcomes of the app users’ infants from birth to 12 months of age. The direct effects on parenting outcomes have been reported separately [24]. It was hypothesized that infants in the intervention group would exhibit better language, motor, cognitive, and social skills than their counterparts in the control group.

## Methods

### Study Design

A 2-group parallel prospective longitudinal design was adopted for this study, which was conducted from February 2020 to July 2022. Expecting parents were recruited from 2 public health care institutions in Singapore. The study was part of a randomized controlled trial (RCT) investigating the effectiveness of SPA in improving perinatal parental outcomes such as postnatal depression and anxiety [24]. Along with their parents, the infants in this study were randomly allocated to either the SPA intervention group or standard care control group.

### Eligibility Criteria

Parents were considered eligible for the study if they met the following criteria: (1) both parents were aged ≥21 years; (2) both parents were able to read and speak English; (3) the pregnancy was at low risk with >24 weeks of gestation (age of viability in Singapore); and (4) both parents owned a smartphone with internet access. Parents were excluded from the study if they had high-risk pregnancies (eg, pregnancy-induced hypertension, preeclampsia, and placenta previa major). Infants who were born via a complicated assisted delivery where the mother required prolonged hospitalization and admitted to the neonatal intensive care unit and infants with congenital issues were excluded from the study to minimize confounding influences on the outcome variables.

### Sample Size Calculation

As this study was part of an RCT investigating the effectiveness of the SPA intervention on parental outcomes, the parents enrolled in the RCT and their infants were recruited for this study. Considering the medium-sized effect of SPA, a Cohen *d* of 0.5 (90% power and 0.05 significance), and an attrition rate of 20% (based on another study) [25], 200 couples were recruited for the main RCT. Two couples had twins; therefore, 202 infants were recruited for this study.

### Intervention

The control group parents received the standard perinatal care offered by the hospitals they were recruited from, which consisted of antenatal checkups, optional antenatal classes, care during their stay in the ward, and a postnatal review scheduled 6 weeks post partum. Perinatal care was provided to the parents by obstetricians, nurses, neonatologists, and lactation consultants. The intervention group parents received the standard perinatal care as well, but they were also granted access to the mHealth intervention SPA upon recruitment into the study. In addition, they were matched with trained peer volunteers, who were experienced mothers trained by the research team to provide peer support for the parents in the RCT.

SPA included a variety of pregnancy-, childbirth-, postpartum-, and infant care–related information. This included articles, audio files, and videos about birth preparation, bonding and attachment across the perinatal period, breastfeeding, baby care–related tasks (from bathing to safe sleep habits), and involvement of both fathers and mothers in baby care tasks. The information was curated by the health care professionals involved in the study so that parents could conveniently access reliable and accurate information. Expert advice, discussion forums, and frequently asked questions were also features of the mobile app that aimed to resolve any pregnancy- or childcare-related queries that the parents might have. The parents were encouraged to interact with the peer volunteer with whom they were matched if they needed emotional or informational support from experienced mothers who had previously had and recovered from postnatal depression. Detailed features of the SPA mobile app and peer volunteer intervention can be found in the published development study [26]. The SPA intervention was made available to the intervention group parents from the point of recruitment until 6 months post partum.

### Procedure

Couples were recruited by a research assistant during their scheduled antenatal checkups at 2 tertiary hospitals in Singapore. They were provided with an explanation of the study, and interested couples were screened for eligibility before giving them an informed consent form where they could indicate their willingness to participate in the study. Subsequently, the couples were randomly allocated to the intervention or control group. The estimated due date of the couples was recorded, and the couples were then contacted shortly after their due date to gather information regarding their childbirth (eg, gender of the baby and whether they attended prenatal classes). The parents also entered this information into SPA so that the app could send them information that is specific and relevant to the infant’s age and respective postpartum time points.

The parents were contacted via SMS text messages to complete the follow-up questionnaires at 1, 2, 4, 6, 9, and 12 months post partum. Mothers tended to be the ones who completed the infant-related questionnaires. A house visit was also scheduled at 6 and 12 months, during which a trained research assistant visited the participants’ homes to assess the infant using the Bayley-4.

### Outcome Measures

Conducting research with very young children involves various challenges regarding the accuracy of the data collected, as infants are not verbal, and thus it is difficult to obtain information directly from them. Therefore, the following instruments were used to measure the constructs examined in this study to provide an accurate representation of the infants’ developmental progress.

#### Ages and Stages Questionnaire—Third Edition

The parent-reported Ages and Stages Questionnaire (ASQ) was used to measure the development of the infants across 5 domains: personal-social, gross motor, fine motor, problem solving, and communication [27]. There are 21 sets of ASQ, each catering to a different developmental time point; these can be used to assess infants or children aged 2 to 66 months. Existing literature has found that Cronbach α for the ASQ ranges from .49 to .87, depending on the domain and time point [28]. In this study, the ASQ sets were administered at 2, 4, 6, 9, and 12 months. Each set of ASQ consisted of 30 items, and the parents were asked to select “yes” (10 points), “sometimes” (5 points), or “not yet” (0 points) to indicate whether their child had demonstrated the milestone described in that particular item. Cutoff scores, which were 2 SDs below the normative mean, were provided to indicate whether the infant’s development required further monitoring and assessment (Table 1). Cronbach α for the ASQ sets administered in this study are also presented in Table 1.

**Table 1:** Cronbach α and cutoff scores for the Ages and Stages Questionnaire.

			2 months		4 months		6 months		9 months		12 months
Cronbach α			.746		.883		.864		.877		.910
**Cutoff scores^a^**											
	Communication	22.77		34.60		29.65		13.97		15.64	
	Gross motor	41.84		38.41		22.25		17.82		21.49	
	Fine motor	30.16		29.62		25.14		31.32		34.50	
	Problem solving	24.62		34.98		27.72		28.72		27.32	
	Personal-social	33.71		33.16		25.34		18.91		21.73	

^a^Scores below the cutoff indicate that the child could be at risk of neurodevelopmental conditions, and further assessment with a professional might be needed.

#### Bayley Scales of Infant and Toddler Development—Fourth Edition

The Bayley-4 consists of 5 scales: cognitive, language, motor, social-emotional, and adaptive behavior (ADBE) [29]. The assessment was administered by a research assistant involved in the study and scored based on the research assistant’s observation of the infant’s performance in various tasks. The Bayley-4 assessment was conducted at 6 and 12 months post partum, and the number of items administered varied depending on the infant’s performance. The Bayley-4 items were scored on a 3-point Likert scale ranging from 0 to 2. Points were added to form a total raw score, which could be converted into scaled or standard scores. Cutoff scores were also provided, where standard scores <85 were marked as “at-risk” to indicate possible developmental delay [30]. Prior studies [31,32] that administered Bayley-III assessments have reported Cronbach α ranging from .88 to .96 across all scales. Cronbach α of the newest Bayley-4 assessment has not been reported in the existing literature; in this study, the average Cronbach α of the Bayley-4 was found to be .61.

#### Brief Infant Toddler Social Emotional Assessment

The 42-item parent-reported Brief Infant Toddler Social Emotional Assessment (BITSEA) was divided into 2 scales: the problem scale (31 items) and the competence scale (11 items) [33]. The BITSEA items were scored on a 3-point Likert scale ranging from 0 to 2. The scores for the items in each scale were added to obtain the respective total score. Higher scores on the problem scale indicated a higher frequency and range of behavioral and emotional problems, whereas higher scores on the competence scale indicated a higher level of social competence. For the competence scale, the cutoff score was 11, whereas for the problem scale, the cutoff score was 13 for girls and 12 for boys. The BITSEA was administered only during the 12-month follow-up, as it is meant to be administered to infants from 12 months onward. The Cronbach α for the BITSEA was .71, similar to that in a previous study [34], where the Cronbach α for the problem and competence scales were .82 and .72, respectively.

### Data Analysis

Data analyses were conducted using SPSS (version 27.0; IBM Corp) [35], and statistical significance was set at *P*<.05. Descriptive statistics are presented as mean (SD) for continuous variables and as n (%) for categorical variables. Linear regression was used to examine the association between continuous outcome scores and the intervention adjusted for baseline measures and other covariates. Each participant’s score on the 3 instruments was subsequently categorized based on the cutoff scores determined by the respective developers of each instrument. Modified Poisson regression was used to analyze the association between binary outcome scores and the intervention adjusted for baseline measurements and other covariates based on the cutoff scores for each instrument. This was done to compare the proportion of infants in each group who fell into the at-risk category for each domain. Established correlations between the main outcomes and covariates based on previous studies [36,37] were used to determine which covariates were needed to be statistically corrected for.

### Ethics Approval

Before the commencement of the study, ethics approval was obtained from the National Health Group Domain Specific Review Board (NHG DSRB:2019/00875). The parents of the infants involved in this study were provided with information on the study and its procedures before they provided their written consent. It was communicated to the parents that participation was voluntary and that they had the right to withdraw anytime without incurring any consequences.

## Results

### Overview

In total, 200 couples and their infants were recruited for this study. However, owing to the attrition rate of 28.5% that was reported in the main RCT [24], only 79% (158/200) of infants were included in the analysis (the remaining 42/200, 21% parent-infant dyads dropped out of the study). The demographic characteristics of the participants are presented in Table 2. The mean age of the parents was 31.4 (SD 4.93) years, and Malay (115/316, 36.4%) and Chinese (125/316, 39.6%) were the most common ethnicities. Most (87/316, 55.1%) of the infants were male. Most (257/316, 81.3%) parents did not attend any prenatal courses.

**Table 2:** Demographic characteristics of the parents and their infants.

Demographic characteristics			Intervention group	Control group
**Age of parents, mean (SD)**				
	Mothers		29.9 (4.2)	30.5 (4.2)
	Fathers		32.1 (4.9)	33.3 (5.4)
**Parent’s ethnicity (intervention: n=83; control: n=75), n (%)**				
	**Mothers**		83 (100)	75 (100)
		Chinese	31 (37.3)	28 (37.3)
		Malay	32 (38.6)	27 (36)
		Indian	14 (16.9)	9 (12)
		Others	6 (7.2)	11 (14.7)
	**Fathers**		83 (100)	75 (100)
		Chinese	35 (42.2)	31 (41.3)
		Malay	29 (34.9)	27 (36)
		Indian	14 (16.9)	10 (13.3)
		Others	5 (6)	7 (9.3)
**Sex of baby (intervention: n=86; control: n=72), n (%)^a^**				
	Male		44 (51.2)	43 (59.7)
	Female		42 (48.8)	29 (40.3)
**The educational level of parents (intervention: n=83; control: n=75), n (%)**				
	**Mothers**		83 (100)	75 (100)
		Primary school	0 (0)	0 (0)
		Secondary school	11 (13.3)	4 (5.3)
		ITE^b^, polytechnic, or junior college	25 (30.1)	35 (46.7)
		University	47 (56.6)	36 (48)
	**Fathers**		83 (100)	75 (100)
		Primary school	0 (0)	2 (2.7)
		Secondary school	17 (20.5)	7 (9.3)
		ITE, polytechnic, or junior college	27 (32.5)	34 (45.3)
		University	39 (47)	32 (42.7)
**Monthly household income (SGD $; intervention: n=164; control: n=147), n (%)^c^**				
	<1000 (<US $761.88)		14 (8.5)	5 (3.4)
	1000-3000 (US $761.88-$2285.64)		34 (20.7)	40 (27.2)
	3000-5000 (US $2285.64-$3809.39)		50 (30.5)	47 (32.0)
	>5000 (>US $3809.39)		66 (40.2)	55 (37.4)
**Attended prenatal courses (intervention: n=166; control: n=150), n (%)**				
	Yes		33 (19.9)	26 (17.3)
	No		133 (80.1)	124 (82.7)

^a^Only 158 infants (86 in the intervention group and 72 in the control group) were included in the analysis because 42 parent-infant dyads dropped out of the study by 6 months post partum.

^b^ITE: Institute of Technical Education.

^c^Not all parents provided this information.

### Communication

The mean and SD scores for the ASQ and Bayley-4 are presented in Tables 3 and 4, respectively, along with the proportion of infants who scored below the cutoff scores (labeled as “at-risk”). Results from the generalized linear regression model indicated that the infants in the intervention group scored significantly higher on the communication domain of the ASQ at 6 (effect size=3.31, 95% CI 0.10-6.53; *P*=.04) and 9 (effect size=6.14, 95% CI 0.90-11.38; *P*=.02) months post partum. However, this difference was not significant after the Bonferroni adjustment (at 4 months: *P*=.22; at 6 months: *P*=.11). The Poisson regression results showed that the intervention group infants were less likely to fall under the at-risk category, but there were only a few at-risk cases (Table 3); therefore, the estimation might not be reliable. Results from the linear (effect size=9.304, 95% CI 5.58-13.13; *P*<.001) regression model of the 12-month Bayley-4 assessment also showed that the infants from the intervention group tended to perform better than those from the control group on the language scale.

**Table 3:** Ages and Stages Questionnaire scores based on domains.

		2 months (n=158)			4 months (n=146)			6 months (n=143)			9 months (n=146)			12 months (n=140)	
		Intervention (n=87)	Control (n=71)	Intervention (n=83)		Control (n=63)	Intervention (n=76)		Control (n=67)	Intervention (n=81)		Control (n=65)	Intervention (n=78)		Control (n=62)
**Communication**															
	Values, mean (SD)	48.72 (12.16)	48.68 (12.97)	53.35 (7.83)		53.11 (8.69)	50.47 (7.54)		47.31 (9.52)	43.38 (14.82)		37.71 (13.56)	48.03 (11.53)		46.11 (13.09)
	At risk^a^, n (%)	4 (4.7)	4 (5.6)	1 (2)		1 (2.7)	0 (0)		1 (1.9)	2 (2.9)		2 (4.2)	1 (1.5)		2 (4.4)
**Gross motor**															
	Values, mean (SD)	52.38 (11.50)	49.65 (10.76)	54.02 (8.78)		50.41 (10.30)	43.26 (11.82)		41.11 (14.90)	42.57 (16.10)		41.35 (17.59)	48.64 (13.60)		48.11 (12.58)
	At risk, n (%)	11 (12.8)	15 (20.8)	4 (7.8)		5 (13.5)	4 (6.1)		5 (9.6)	6 (8.8)		5 (10.4)	2 (3)		2 (4.4)
**Fine motor**															
	Values, mean (SD)	47.56 (10.28)	45.49 (10.08)	46.47 (14.36)		45.00 (13.54)	45.85 (12.52)		42.12 (15.91)	46.91 (13.74)		45.00 (12.68)	46.97 (13.21)		49.00 (11.01)
	At risk, n (%)	10 (11.6)	8 (11.1)	6 (11.8)		3 (8.1)	5 (7.6)		11 (21.2)	12 (17.6)		6 (12.5)	10 (15.2)		4 (8.9)
**Problem solving**															
	Values, mean (SD)	43.08 (15.93)	43.75 (12.58)	48.63 (12.77)		48.47 (11.52)	45.77 (12.75)		60.10 (138.72)	42.65 (14.36)		41.28 (12.75)	40.23 (15.60)		40.22 (15.11)
	At risk, n (%)	11 (12.8)	4 (5.6)	8 (15.7)		5 (13.5)	7 (10.6)		9 (17.3)	9 (13.2)		9 (18.8)	14 (21.2)		12 (26.7)
**Personal-social**															
	Values, mean (SD)	48.60 (10.62)	47.57 (10.45)	49.12 (10.43)		48.38 (11.49)	45.62 (11.84)		43.65 (12.88)	37.57 (12.77)		34.26 (12.51)	38.38 (15.74)		37.33 (15.90)
	At risk, n (%)	7 (8.1)	8 (11.1)	4 (7.8)		4 (10.8)	5 (7.6)		7 (13.5)	3 (4.4)		4 (8.3)	12 (18.2)		9 (20)

^a^The at-risk group refers to infants who scored below the cutoff scores stated in Table 1.

**Table 4:** Mean and SD of the Bayley-4 standard scores based on domains.

		6 months (n=109)		12 months (n=105)	
		Intervention (n=59)	Control (n=50)	Intervention (n=55)	Control (n=50)
**Cognitive**					
	Values, mean (SD)	100.76 (9.64)	95.12 (15.59)	98.09 (10.34)	89.52 (10.41)
	At risk^a^, n (%)	3 (5.1)	6 (12)	3 (5.5)	10 (23.8)
**Motor**					
	Values, mean (SD)	—^b^	—	100.18 (10.36)	98.64 (10.76)
	At risk, n (%)	—	—	5 (9.1)	8 (19)
**Language**					
	Values, mean (SD)	—	—	95.42 (7.87)	93.02 (6.91)
	At risk, n (%)	—	—	4 (7.3)	7 (16.7)
**Social-emotional**					
	Values, mean (SD)	—	—	97.09 (20.68)	100.00 (18.35)
	At risk, n (%)	—	—	15 (27.3)	7 (16.7)
**Adaptive behavior**					
	Values, mean (SD)	99.88 (9.20)	96.96 (9.43)	98.18 (8.71)	96.10 (8.57)
	At risk, n (%)	3 (5.1)	4 (8)	3 (5.5)	4 (9.5)

^a^The at-risk group refers to infants with standard scores <85.

^b^Data not available.

**Figure 1:**
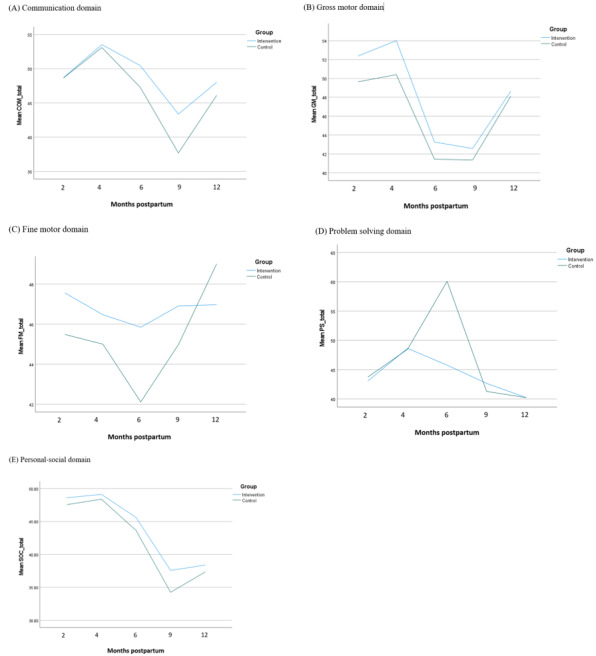
Trend graphs for the changes in the Ages and Stages Questionnaire scores over time: (A) communication domain, assessing speech and language; (B) gross motor domain, assessing ability to produce large movements; (C) fine motor domain, assessing smaller movements; (D) problem solving domain, assessing cognitive and intellectual skills; (E) personal-social domain, assessing emotional and social skills.

Figure 1A shows the changes in the ASQ scores in the communication domain from 2 to 12 months post partum in both groups. The infants from both groups showed similar trends: there was an initial increase in communication scores at 2 and 4 months post partum, and, subsequently, there was a steep decrease in communication scores from the 4- to 9-month time points before they increased again at 12 months post partum. From 4 months onward, the infants in the intervention group scored higher in the communication domain than those in the control group. The largest difference was observed at 9 months post partum. Overall, the infants from the intervention group demonstrated better communication skills than those from the control group.

### Motor Skills

The infants from the control group were significantly more likely to score below the cutoff score of the ASQ gross motor domain at 2 months post partum (risk ratio [RR]=0.417, 95% CI 0.20-0.85; *P*=.02). Although the infants from the intervention group tended to score higher than those from the control group in the gross motor domain (Table 3), this difference was not significant (*P*=.71).

The intervention group infants were found to have better fine motor skills than the control group infants at 6 months post partum, based on the results of the logistic regression model analysis. The infants from the control group were more likely to score below the cutoff score on the ASQ fine motor domain than those from the intervention group (RR=0.25, 95% CI 0.08-0.76; *P*=.02). In addition, the infants from the intervention group had significantly higher scores on the ASQ fine motor domain than those from the control group (effect size=6.02, 95% CI 1.03-11.02; *P*=.02). However, there were no significant differences between both groups in the Bayley-4 motor scale scores at 12 months post partum (effect size=−4.91, 95% CI −12.96 to 3.14; *P*=.23).

Similar to the trend graph of the ASQ communication domain, the ASQ gross motor graph showed that the intervention group scored better than the control group on the gross motor items (Figure 1B). The gross motor scores of both groups slightly increased during the first 2 time points before sharply decreasing at 6 months post partum. There was a steady increase in the gross motor scores of both groups from 9 to 12 months post partum, accompanied by a decreasing difference in gross motor scores. Figure 1C shows the trend graph of the ASQ fine motor scores. The fine motor scores of both groups of infants reduced from 2 to 6 months post partum before gradually increasing again. At 12 months post partum, the infants from the control group had higher scores than those from the intervention group, but this difference was not significant (effect size=−0.98, 95% CI −5.87 to 3.91; *P*=.69).

### Cognition

At 6 months post partum, the infants from the control group had a higher chance of being in the at-risk group for the problem solving domain of the ASQ than those from the intervention group (RR=0.34, 95% CI 0.12-0.91; *P*=.03). However, the linear regression model did not find any significant differences in problem solving scores between the 2 groups (effect size=−16.88, 95% CI −52.34 to 18.59; *P*=.35). The infants from the intervention group fared significantly better than their control group counterparts on the cognition scale of the Bayley-4 assessment (effect size=9.30, 95% CI 5.48-13.12; *P*<.001) at 12 months post partum.

According to Figure 1D, the infants from the control group generally scored higher in the problem solving domain than those from the intervention group. There was a sharp increase in the problem solving scores of the control group infants at the 6-month time point. Following this increase, an equally sharp decrease in problem solving scores was found at 9 months post partum, where the control group scores fell below those of the intervention group.

### Social-Emotional Skills

No significant group differences were found in the scores related to the social-emotional skills of the infants across all time points (at 2 months: *P*=.28; at 4 months: *P*=.61; at 6 months: *P*=.17; at 9 months: *P*=.06; at 12 months: *P*=.57). This was true for the personal-social domain of the ASQ, the social-emotional scale of the Bayley-4 assessment, and the competence scale of the BITSEA.

Figure 1E shows the changes in the ASQ personal-social scores of the infants from both groups. Scores on the personal-social domain were relatively high during the 2- and 4-month time points but decreased from 4 to 9 months post partum. The personal-social scores increased subsequently at 12 months post partum. Overall, the intervention group appeared to perform better in terms of social-emotional skills than the control group.

### Behavioral Outcomes

The ADBE scale of the Bayley-4 and the problem scale of the BITSEA assessed the behavioral outcomes of the infants. The BITSEA problem scale covered externalizing behaviors, dysregulating behaviors, and maladaptive behaviors. The infants from the control group were found to have significantly higher scores on the BITSEA problem scale than those from the intervention group (effect size=−5.87, 95% CI −10.44 to −1.70; *P*=.006). As such, the control group infants tended to exhibit more problem behaviors, as described in the BITSEA. By contrast, the ADBE scale mainly focused on the infants’ ability to engage in functional developmental tasks that are critical to their survival. This included feeding oneself and communicating basic needs. No significant group differences in the ADBE scores were found at both 6 months (effect size=2.05, 95% CI −2.74 to 6.84; *P*=.40) and 12 months (effect size=0.954, 95% CI −2.53 to 4.44; *P*=.59) post partum.

### Analysis of Covariates

Parents attending prenatal courses was found to significantly influence whether their infants’ ASQ scores on the communication (RR=0.13, 95% CI 0.02-0.75; *P*=.01), gross motor (RR=0.43, 95% CI 0.21-0.90; *P*=.03), and personal-social (RR=0.28, 95% CI 0.10-0.82; *P*=.02) domains fell below the cutoff at 2 months post partum. The infants whose parents had attended prenatal courses tended to score better in these domains.

The education level of the parents was also a predictor of the infants’ motor skills. The infants of parents with secondary educational qualifications had a higher chance of being in the at-risk group at 6 months post partum than those of parents who graduated from universities (RR=13.41, 95% CI 2.27-79.09; *P*=.004). The generalized linear regression model for fine motor skills at 6 months post partum also showed that the infants of parents who received up to secondary-level education scored significantly lower than those of parents who graduated from universities (effect size=−13.55, 95% CI −24.95 to −2.15; *P*=.02). Thus, the results suggest that the infants of parents with higher educational levels tended to have developed better motor skills at 6 months post partum.

In this study, monthly household income was found to significantly affect the cognition and motor skills of the infants. The infants from households with a monthly income between SGD $3000 (US $2285.64) and SGD $5000 (US $3809.39) were more likely to belong to the at-risk group of the cognition domain of the Bayley-4 assessment than those from households earning >SGD $5000 (US $3809.39) monthly (RR=14.79, 95% CI 4.96-44.15; *P*<.001). Those with higher monthly household income also scored higher on the motor skills domain of the Bayley-4 assessment at 12 months post partum. Those with household income >SGD $5000 (US $3809.39) per month scored significantly higher in the motor skills domain than those with a household income of SGD $3000 (US $2285.64) to SGD $5000 (US $3809.39) per month (effect size=−10.65, 95% CI −15.42 to −5.89; *P*<.001) and those with a household income <SGD $1000 (US $761.88) per month (effect size=−14.97, 95% CI −22.97 to −5.17; *P*=.002). The generalized linear regression model also found similar results with the gross motor scale of the ASQ (effect size=−9.91, 95% CI −15.76 to −4.06; *P*<.001). Nonetheless, the modified Poisson regression model did not find any significant differences in the number of infants at risk for delayed motor skill development based on their income groups (*P*=.68).

## Discussion

### Principal Findings

This study examined the effects of the SPA intervention on infants’ developmental outcomes during the first 12 months of life. The infants in the intervention group mostly scored better in domains assessing communication, cognition, and social-emotional development. More infants from the control group fell under the at-risk category for motor skills than those from the intervention group. Findings from the main RCT reported a high attrition rate of 28.5% [24]. The outbreak of the COVID-19 pandemic in early February 2020 may have affected the parents’ and infants’ participation in the study, especially for the home visits. Hence, this led to a smaller-than-expected sample size, which likely affected the statistical power of the study. In general, although the infants from the intervention group tended to exhibit better developmental outcomes than those from the control group, these differences were modest. Therefore, the results of this study are not completely in line with the hypothesis.

### Communication

From 2 to 12 months post partum, the infants from the intervention group were found to exhibit better communication skills than their control group counterparts. According to Bortfeld and Gabouer [38], early infant communication lays the groundwork for language. Previous studies [38,39] have emphasized that communication development often begins in the womb, where the fetus receives auditory inputs that enable them to start learning how to distinguish sounds. The SPA knowledge base included content encouraging the intervention group parents to communicate with their newborn and suggested ways to enhance parent-child interactions, even during pregnancy. For example, expecting parents can respond to the kicks made by the fetus during late pregnancy to communicate with them. With newborns, parents can play soothing music or read stories aloud to them to facilitate better communication and language development. According to the SPA traffic data, many parents in the intervention group accessed these materials in the mobile app and thus communicated with their infants more effectively, boosting their communication development. The decrease in communication scores from 4 to 9 months in both groups was unsurprising, as normative scores on the ASQ vary across ages and domains. For communication, the normative scores were approximately 42.5 at 4 months, 34 at 6 months, and only 30 at 9 months. Therefore, the reduction in mean communication scores during this period does not indicate that the infants’ communication abilities did not progress during this period. They mostly performed well above the normative scores.

On the basis of the trend graphs, the difference in the communication scores between the 2 groups increased over time. Topping et al [40] explained that parent interaction during the infant’s preliteracy development was important in enhancing the child’s future language abilities. Parenting intervention programs have been found to have a positive impact on children’s language development. It is possible that the intervention group parents acted upon the things that they had read about, such as child play and infant milestones, which enabled them to interact more with their newborns. This, in turn, would have enhanced their child’s future language development. Therefore, the implementation of the SPA intervention might have led to the widening difference in communication scores between the 2 groups from 6 months onward. Future research could investigate in greater detail the relationship between the materials that parents read and what they put into practice.

### Motor Skills

Similar to the communication trend graphs, the fluctuations in gross and fine motor skills indicated by the ASQ scores can be attributed to the differences in normative scores. Overall, the motor skills of the intervention group infants developed to a greater extent than those of the control group infants. This might have been because the parents in the intervention group had read the guide on how they could engage in play with their children on SPA. The guide included some toy recommendations that can help improve the motor skills of infants by allowing them to practice movements such as grasping and head turning. Semistructured interviews with some of the parents [10] revealed that the parents enjoyed having access to SPA, as it included various localized information that applied to them, which was unique because other parenting apps were more general. The parents could also anticipate and encourage the growth of their infants, as they read about developmental milestones. Consequently, the infants from the intervention group were exposed to more opportunities to practice and develop their motor skills. Descriptions of developmental milestones were also provided to educate parents on the motor skills that their children should achieve at each stage of development. However, the results suggest that the positive effects of the SPA intervention on the infants’ motor skills did not persist beyond 6 months post partum. As the SPA intervention only lasted up to 6 months post partum, information related to infant motor skill development beyond 6 months was rather scarce. The achievement of various motor-related milestones often drastically changes the subsequent behaviors of infants; thus, the toys or games used to engage them during earlier months might be less relevant in facilitating more advanced motor skill development [2]. Further research is needed to determine whether more age-appropriate information on motor skill development would help improve the motor skills of infants aged >6 months.

### Cognition

Results from the problem solving domain of the ASQ revealed that the control group infants generally fared better than the intervention group infants during the first 6 months of their lives. However, the results of the 12-month Bayley-4 assessment revealed that the intervention group infants did better on the cognition scale than the control group infants. This finding is contrary to the hypothesis of this study, which proposed that the intervention group infants would perform better on cognition tasks than the control group infants. A reason for this might be that SPA did not include much content on enhancing the cognition and problem-solving skills of infants. Most of the parenting information available was related to childcare tasks such as feeding and swaddling or was related to parent-child communication and motor skills. As reported in a published qualitative paper [10] regarding the perspectives of the parents in the study, the control group parents described how they took the initiative to explore web-based resources and installed other parenting mobile apps to obtain more parenting-related information than what the standard care offered [10]. Some mobile apps used by the control group parents might have included information on how to build on the cognitive development of the infants during the first few months post partum. Therefore, more research is needed to obtain greater insight into the parenting resources used by Singaporean parents and how they influence infant development. More importantly, health care providers developing educational programs such as SPA should consider including content focusing on the holistic development of infants and children.

### Social-Emotional Skills

Although there were no statistically significant group differences in the personal-social domain of the ASQ and social-emotional scale of the Bayley-4 assessment, the intervention group generally demonstrated greater social-emotional development. This lack of significance could be attributed to the fact that SPA did not include much information regarding the development of social-emotional skills in infants. However, the encouragement provided to parents to engage in age-appropriate parent-child play and increase parent-child interactions might have contributed to the slightly higher social-emotional and personal-social scores [38]. As mentioned, the COVID-19 pandemic hit Singapore in early 2020, immediately after the study began. During this period, various restrictions were set in place to minimize social interactions to prevent the spread of the virus [41]. This included the closure of infant care and prohibition of social gatherings. Hence, there were fewer opportunities for infants to engage in social situations with other infants and foster peer relationships. Existing literature has found that playing with peers is an important activity that allows for better development of prosocial behaviors and the formation of relationships with others [42]. The lack of such interactions owing to the pandemic might have further undermined the significance of the group difference in social-emotional development.

### Behavioral Outcomes

The infants from the intervention group were found to engage in more ADBEs than those from the control group. On the basis of the parents’ responses to the BITSEA, the control group infants exhibited more problem behaviors as well. Maternal responsivity and sensitivity to infant distress are important factors in predicting ADBE in infants. Higher maternal responsivity is associated with greater emotional regulation and fewer behavioral issues [43]. Lorber et al [44] also found that well-known predictors of externalizing behavior include daily parenting hassles, authoritarian parenting, and poor parent-child bonding. The parents who received the SPA intervention were more educated on how to interact and bond with their newborns, which possibly led to improved parent-child bonding. They were also provided with support from the peer volunteers, which helped reassure them and provide them with an avenue to discuss parenting-related worries [10]. Ultimately, the parents in the intervention group received more informational, appraisal, and emotional support than those in the control group [10,26]. This helped them better adjust to the newborn care tasks that they had to take on after childbirth. Therefore, this study suggests that the SPA intervention was effective in facilitating the development of ADBEs in infants.

### Prenatal Courses, Education, and Household Income

This study found that prenatal classes had a positive impact on communication, gross motor, and personal-social scores at 2 months post partum. Prior research [45] has found that prenatal education courses can help reduce anxiety during pregnancy, which can help improve prenatal bonding. This is especially true for first-time parents, who experience greater fear of childbirth and parenting self-efficacy [46]. Improved maternal well-being would then facilitate better infant outcomes, such as fewer maladaptive tendencies and greater levels of social competence [47]. However, most of the parents in this study did not attend prenatal classes. During interviews, the parents revealed that they were unable to attend these classes, as they were canceled because of the COVID-19 pandemic [10]. This left many new parents unprepared for the transition to parenthood, causing them to feel stressed and clueless. The implementation of social distancing restrictions also amplified this problem, as parents were unable to seek instrumental support from their family members or nannies [10]. Therefore, it is crucial for maternal care institutions to prepare and provide parents with sufficient support, especially through remote means at times when the availability of support is affected. This study was unable to examine how the development of children of new parents and that of children of experienced parents differed, as information regarding whether the parents were new or experienced was not collected in the main RCT. Therefore, it is crucial for future research to gather this information to further examine how the intervention impacts the differing needs of these parents.

The infants from families with higher household incomes were found to have more developed cognition. This is supported by previous literature, where it was found that children from families with low socioeconomic status (SES) had lower cognitive flexibility [48]. It was explained that the consequences of living with low SES are less favorable for children’s development. This includes greater exposure to stress, which affects children’s performance on cognitive tasks, and reduces maternal sensitivity and verbal stimulation [48]. Unsafe living conditions and stressors associated with low SES may also result in more negative or authoritarian parenting, which can affect cognitive outcomes.

Both higher parental education and higher monthly household income were significantly associated with stronger motor skills. Freitas et al [49] evaluated the relationship between SES and the availability of resources to promote motor development in infants. This study found that SES is a crucial factor influencing the availability of motor affordances at home. Educational level was also found to significantly affect the provision of toys to infants [49]. Parental education often affects the SES of the family, as higher education levels tend to open up job opportunities that offer higher income. Having more income would then lead to the purchase of more play materials or even the ownership of a larger home that provides more physical space for motor development [49,50]. As a result, infants from low-SES families and infants of parents with lower education levels tend to develop motor skills more slowly. Hence, researchers, health care providers, and policy makers may focus their efforts on developing interventions focusing on family factors that contribute to infant developmental outcomes across SES.

### Strengths and Limitations

Given that social support has been proven to improve parental well-being and, in turn, promote infant growth in various areas, the SPA intervention was developed. The intervention aimed to meet the support needs of Singaporean parents during the perinatal period, thus helping them adjust to parenting roles and infant-care tasks. This study found that technology-based parenting interventions such as SPA can lead to benefits beyond enhancing parental well-being. The findings of this study are crucial for the future development of not only mobile apps for parents in Singapore but also those for parents in other countries. Providing parenting education and emotional support can indirectly improve infant developmental outcomes. However, it is important to recognize and consider the cultural beliefs, practices, and support needs of parents from other countries. This would allow app developers to provide a knowledge base and appraisal support that would be respectful of and helpful in meeting their individualized needs.

Another strength of this study is that it used questionnaires that were completed by both parents and trained personnel. Parent-completed measures are advantageous in that parents spend the most time with their infants and are the most knowledgeable about them. However, existing literature [51] has also pointed out that there are biases associated with parent-reported questionnaires. Generally, parents are not trained in evaluating the development of infants, which can make them susceptible to overestimating or underestimating their child’s abilities and thus render their responses less reliable. By contrast, although the Bayley-4 was administered by a trained research assistant and can, therefore, provide a more objective assessment of the infant’s growth, infants tend to behave differently with unfamiliar individuals [51]. As such, Miller et al [51] expressed that a fuller picture of the infant’s development could be obtained if both types of assessments were used.

This study has some limitations. One of its limitations is its high attrition rate. Because of the COVID-19 pandemic, parents were more cautious of physical interactions, as they did not want themselves, their infants, or other family members to contract the virus. Therefore, many parents declined home visits for the Bayley-4 assessment. This may have affected the accuracy of the findings in representing the sample recruited for this study. Moreover, the longitudinal nature of the study might have also contributed to the high attrition rate. Parents tend to become busier post partum owing to the need for them to adjust to parenting responsibilities; in the case of this study, the need to take extra precautions to prevent contracting the COVID-19 virus added to these responsibilities. Therefore, it is paramount to devise strategies to keep parents interested in and willing to participate in the study. For example, research team members can frequently contact parents to build stronger rapport and remind them to access SPA if they have parenting-related concerns. Although the research team originally intended to do so, some technical issues led to the absence of chat notifications, affecting the communication between the team and parents. Furthermore, many of the research team members were also frontline health care workers; therefore, they were unable to meet often and resolve these issues in a timely manner.

Another limitation of this study is the lack of information regarding whether the parents were experienced or new. This is an important limitation, as the struggles and support needs that new and experienced parents encounter may differ widely. Hence, future studies should take note to collect such information from parents to provide deeper perspectives regarding the effectiveness of parenting interventions in new and experienced mothers and fathers. Subsequent research may also investigate parental sensitivity and responsivity to provide further insight into how they may affect infant behavioral outcomes.

### Conclusions

This study examined the effects of the SPA intervention on infant developmental outcomes. The results showed that the infants from the intervention group generally developed better in terms of communication, motor skills, cognition, and social-emotional skills than those from the control group. The peer support and informational support that the SPA intervention offered to the intervention group parents were thus helpful in indirectly influencing the development of infants. More research is needed to obtain an in-depth understanding of what functions of the intervention influenced the infant outcomes and what information the current generation of parents hopes to see in parenting mobile apps. This would facilitate the creation of more effective mHealth app–based support for parents. In the future, interventions targeting infant growth and development should be created to measure the direct effects of educational interventions. In addition, future parenting interventions should focus on providing more support to families with lower SES to help promote the development of infants and support parents from these families.

## Abbreviations

ADBE: adaptive behavior
ASQ: Ages and Stages Questionnaire
BITSEA: Brief Infant Toddler Social Emotional Assessment
mHealth: mobile health
RCT: randomized controlled trial
RR: risk ratio
SES: socioeconomic status
SPA: Supportive Parenting App
